# The bifunctional SDF‐1‐AnxA5 fusion protein protects cardiac function after myocardial infarction

**DOI:** 10.1111/jcmm.14640

**Published:** 2019-08-30

**Authors:** Fang‐Yang Huang, Tian‐Li Xia, Jun‐Li Li, Chang‐Ming Li, Zhen‐Gang Zhao, Wen‐Hua Lei, Li Chen, Yan‐Biao Liao, Dan Xiao, Yong Peng, Yun-Bing Wang, Xiao‐Jing Liu, Mao Chen

**Affiliations:** ^1^ Department of Cardiology West China Hospital Sichuan University Chengdu China; ^2^ State Key Laboratory of Biotherapy and Cancer Center West China Hospital Sichuan University Chengdu China; ^3^ Laboratory of Cardiovascular Diseases Regenerative Medicine Research Center West China Hospital Sichuan University Chengdu China; ^4^ National Engineering Research Center for Biomaterials Sichuan University Chengdu China

**Keywords:** AnxA5, fusion protein, myocardial infarction, Stromal cell‐derived factor‐1, tissue repair

## Abstract

Stromal cell‐derived factor‐1 (SDF‐1) is a well‐characterized cytokine that protects heart from ischaemic injury. However, the beneficial effects of native SDF‐1, in terms of promoting myocardial repair, are limited by its low concentration in the ischaemic myocardium. Annexin V (AnxA5) can precisely detect dead cells in vivo. As massive cardiomyocytes die after MI, we hypothesize that AnxA5 can be used as an anchor to carry SDF‐1 to the ischaemic myocardium. In this study, we constructed a fusion protein consisting of SDF‐1 and AnxA5 domains. The receptor competition assay revealed that SDF‐1‐AnxA5 had high binding affinity to SDF‐1 receptor CXCR4. The treatment of SDF‐1‐AnxA5 could significantly promote phosphorylation of AKT and ERK and induce chemotactic response, angiogenesis and cell survival in vitro. The binding membrane assay and immunofluorescence revealed that AnxA5 domain had the ability to specifically recognize and bind to cells injured by hypoxia. Furthermore, SDF‐1‐AnxA5 administered via peripheral vein could accumulate at the infarcted myocardium in vivo. The treatment with SDF‐1‐AnxA5 attenuated cell apoptosis, enhanced angiogenesis, reduced infarcted size and improved cardiac function after mouse myocardial infarction. Our results suggest that the bifunctional SDF‐1‐AnxA5 can specifically bind to dead cells. The systemic administration of bifunctional SDF‐1‐AnxA5 effectively provides cardioprotection after myocardial infarction.

## 1 INTRODUCTION

Acute myocardial infarction (AMI) is a severe disease of high mortality and leads to cardiomyocyte loss, scar formation and ventricular remodelling. Although the success of myocardial reperfusion therapy reduces early mortality, the subsequent heart failure is still challenging to treat for the MI survivors.

Chemokine therapy is a promising strategy for myocardial repair.^1^ The most paramount chemokine is stromal cell‐derived factor‐1 (SDF‐1)/CXCL12.^2^ SDF‐1 can protect cardiac function by recruiting stem/progenitor cells^3^ and promoting angiogenesis and cell survival in the infarcted area.^4^, ^5^ Notably, these biological effects depend on a high concentration of SDF‐1 at the local ischaemic area. Previous studies have observed that SDF‐1 is rapidly up‐regulated in heart within minutes to an hour after AMI, but its expression is declining by the following days.^6^ In contrast, the SDF‐1 receptor CXCR4 is expressed later than SDF‐1.^7^, ^8^ Hence, the temporal distinction between SDF‐1 and CXCR4 expression is bound to attenuate the beneficial effects of SDF‐1 on the infarcted myocardium.^9^ It has been attempted to administer exogenous SDF‐1 to rebuild the temporal alignment of SDF‐1 and CXCR4‐positive cells.^9^ However, without the ability to recognize ischaemic tissue, the recombinant SDF‐1 administered via peripheral vein fails to efficiently accumulate at infarcted myocardium. Thus, an efficient delivery system for SDF‐1 is required to produce a local high concentration in the ischaemic area.

Due to the feature that Annexin V(AnxA5) can specifically bind to the exposed phosphatidylserine (PS) of dead cells, AnxA5 has been extensively used as an approach to detect dead cells both in vitro and in vivo.^10^ For instance, it has been reported that the radionuclide labelled AnxA5 can precisely detect dead cells in the infarcted areas.^11^ Meanwhile, our previous study has demonstrated after AnxA5 is attached to cell surface using a biotin‐streptavidin cross‐bridge technique, the modified cells can specifically recognize and bind to dead cells.^12^ However, it remains unclear whether AnxA5 can be used as a protein carrier, especially in the form of fusion protein, to deliver functional proteins to the infarcted myocardium.

Accordingly, the present study was aimed to (a) construct a bifunctional SDF‐1‐AnxA5 fusion protein, (b) verify whether the bispecific fusion protein has the biological functions of both native SDF‐1 and AnxA5 (c) and test the protective effect of SDF‐1‐AnxA5 after mouse myocardial infarction.

## 2 MATERIALS AND METHODS

Methods not described here can be found in Appendix S1.

### 2.1 Cloning of the plasmids encoding the fusion proteins

Primers were designed according to the mature SDF‐1 and AnxA5 cDNA sequences provided by the National Center for Biotechnology Information (GenBank accession no. NM_199168.3 and NM_001154.3). The cDNA sequence for linker (G_4_S)_3_ is produced by gene synthesis (TsingKe biological Technology). SDF‐1, linker and AnxA5 cDNA were cloned from pDONR223‐SDF‐1, pMD19‐Linker and pDONR223‐ANXA5 (both obtained from YouBio) using polymerase chain reaction (PCR) with SDF‐1 primer (sense primer 5′‐CATGCCATGGGGAAGCCCGTCAGCCTG‐3′ and antisense primer 5′‐CTACCTCCGCCACCCTTGTTTAAAGCTTTC‐3′), Linker primer (sense primer 5′‐AAAGCTTTAAACAAGGGTGGCGGAG‐3′ and antisense primer 5′‐GAGAACCTGTGCCATAGAACCGCC‐3′) and AnxA5 primer (sense primer 5′‐ GGCGGTTCTATGGCACAGGTTCTCAG‐3′ and antisense primer 5′ ATGATGATGATGATGGTCATCTTCTCC**AGC** 3′) (the codon mutation is underlined), respectively. The PCR products were washed using DNA Clean Concentrator™‐5 kit according to the manufacturer's protocols. Then, the washed cDNA fragments of SDF‐1, Liner and AnxA5 were mixed and connected using overlap PCR using SDF‐1 sense primer (5′‐CATG**CCATGG**GGAAGCCCGTCAGCCTG‐3′) and AnxA5 antisense primer (5′‐CGC**GGATCC**TTA**ATGATGATGATGATGATG**‐3′; *Nco*I, *Bam*HI restriction sites and 6xHis‐tag sequence were underlined). The PCR products were identified by agarose gel electrophoresis. The SDF‐1‐AnxA5 fusion DNA fragments were digested by restriction enzyme *Nco*I and *BamH*I and inserted into a bacterial expression plasmid pET28a (+). The recombinant plasmid pET28a‐SDF‐1‐AnxA5‐6xHis was confirmed by restriction endonuclease cleavage and DNA sequence. Using a similar approach, we also constructed pET28a‐SDF‐1‐6xHis and pET28a‐AnxA5‐6xHis.

### 2.2 The preparation of recombinant SDF‐1‐AnxA5, native SDF‐1 and AnxA5 proteins

The recombinant plasmids were transformed into BL21(DE3). The bacterial cells harbouring expression plasmids were cultured in the 20 mL LB broth medium with 50 μg/mL kanamycin at 37°C with shaking at 220 rpm for 12 hours. The following day, the bacteria were collected, inoculated into 2 L modified M9CA medium (13.3 g/L M9CA minimum medium, 1 mmol/L MgSO4, 4 g/L Glucose and 2 mg/L Vitamin B1) and grown at 37°C with shaking at 220 rpm to an OD_600_ of 0.5‐0.8. Isopropyl β‑D‑1‑thiogalactopyranoside (IPTG) was added at a concentration of 0.5 mmol/L to induce gene expression for 4 hours with shaking 180 rpm at 30°C. The bacterial cells were collected by centrifugation at 10 000 g for 20 minutes and frozen at −80°C overnight. The bacterial pellets were resuspended in ultrasound buffer (50 mmol/L phosphate buffer, 300 mmol/L NaCl, 10 mmol/L imidazole, 1 mmol/L PMSF, 5 mmol/L DTT and 0.1%Triton X‐100) and disrupted by sonication. The soluble and insoluble fraction were separated by centrifuge to detect protein expression. SDF‐1‐AnxA5 and native SDF‐1 were expressed in the form of inclusion bodies, whereas AnxA5 was in soluble form. Then, the precipitate containing SDF‐1‐AnxA5 and native SDF‐1 was collected after centrifugation at 10 000 *g* for a minute, so was the supernatant containing AnxA5. We performed an on‐column chaperone‐like chemical refolding for inclusion body refolding and purification according to the report of Oganesyan et al^13^. The detailed method of inclusion body protein renaturation was provided in the Appendix S1.

### 2.3 CXCR4 competition assay

Every fluorescence‐activated cell sorting (FACS) tube was prepared with 2 × 10^5^ MOLT‐4 cells and washed with FACS buffer. The MOLT‐4 cells were incubated with the indicated concentrations of the recombinant proteins for 10 minutes at 4°C. Cells were then incubated with anti‐human CXCR4‐PE antibody or isotype IgG2α control antibody for 30 minutes at 4°C. After washing the cells two times with the FACS buffer, cells were analysed using Cytoflex flow cytometry.

### 2.4 Chemotaxis

Transwell migration assays with a pore diameter of 5 μm (for MOLT‐4) or 8 μm (for BMMSCs) were used to detect the chemotaxis. Cells were cultured using a serum‐free medium for 12 hours. A 600 μL amount of recombinant proteins were prepared at the indicated concentrations and added into 24‐well plates. The cells were harvested and resuspended in medium containing 1%FBS at a concentration of 1 × 10^6^/mL (for MOLT‐4) or 1 × 10^5^/mL (for BMMSCs). A 200 μL of cell suspension was added to the upper chamber. The cells were then incubated in CO_2_ incubators. After 4 hours, the number of MOLT‐4 cells in the lower chamber was counted using flow cytometry. After 24 hours, the upper side of the Transwell insert was carefully scratched using a cotton swab. Then, the BMMSCs migrating on the lower side of the Transwell membrane were fixed in methanol for 30 minutes and stained with crystal violet for another 30 minutes at RT. Images of the cells were captured using a microscope. The migrated cells were randomly counted in five microscopic fields.

### 2.5 HUVEC in vitro tube formation assay

Each well of a 96‐well plate was pre‐coated with 50 μL of Matrigel Basement Membrane Matrix with reduced growth factor. HUVECs were harvested and resuspended in endothelial cell medium at a concentration of 3 × 10^5^/mL. One hundred microlitres of the diluted cells were carefully added onto the each Matrigel‐coated well, and then, the SDF‐1‐AnxA5 or native SDF‐1 was added. The endothelial network was viewed under a microscope after 4 hours.

### 2.6 Membrane binding assay

The affinity of fusion protein for cell membranes containing exposing phosphatidylserine was determined by a modification of binding assay of Tait et al^14^ H9C2 cells were injured with hypoxia (5% O_2_, 5%CO_2_ and 90%N_2_) and cultured with glucose‐ and serum‐free medium DMEM for 12 hours. The cells were harvested and resuspended with HEPES buffer containing Ca^2+^. The cell suspensions were incubated with indicated concentrations of recombinant proteins for 5 minutes at 4°C. The commercial Annexin V‐FITC was added to the mixture for further 10‐minute incubation. After incubation, cells were analysed by flow cytometry.

### 2.7 Myocardial infarction model

The investigation conforms to the Guide for the Care and Use of Laboratory Animals published by the US National Institutes of Health (NIH Publication No. 85‐23, revised 1985). Studies using C57BL/6 male mice were approved by the Institutional Animal Care and Use Committee at West China Hospital, Sichuan University. Eight‐ to 12‐week‐old male mice were anaesthetized with a 3% isoflurane, and anaesthesia was maintained throughout the experiment with 1% isoflurane. Mice were placed in the right lateral decubitus position. An oblique incision was made at site 2mm away from the left sternal border, and the heart was exposed through making a 6‐8mm incision in the third intercostal space. With the aid of a surgical microscope, the left anterior descending artery (LAD) could be visualized and permanently ligated with a 7‐0 silk suture at the site of 2 mm below the left appendix.

### 2.8 Administration of SDF‐1‐AnxA5 and G‐CSF

A total of 40 mice were randomly divided into SDF‐1‐AnxA5 group and normal saline group. Among them, 32 survived the surgery and saline (n = 16)/SDF‐1‐AnxA5 (n = 16) administration. Both groups were treated intraperitoneally with G‐CSF (100 μg/g body weight) for consecutive four days. SDF‐1‐AnxA5 (10 μg/g body weight) and normal saline were administered via tail vein immediately and 2, 4, 6 and 14 days after MI established. These mice were killed, and the hearts were harvested 3 days (n = 6 per group), 7 days (n = 5 per group) and 28 days (n = 5 per group) after MI for histological analyses. The experimental design is shown in Figure 5A.

### 2.9 Statistical analysis

Statistical analyses were performed using SPSS statistics software for Windows version 19.0 (IBM SPSS Inc). To compare unpaired groups, we used one‐way ANOVA. The paired data were analysed using a paired t test. Dunnett's Test was used in multiple comparisons. Statistical significance was set at *P* < .05.

## 3 RESULTS

### 3.1 The preparation of SDF‐1‐Annexin V fusion protein

**Figure 1:**
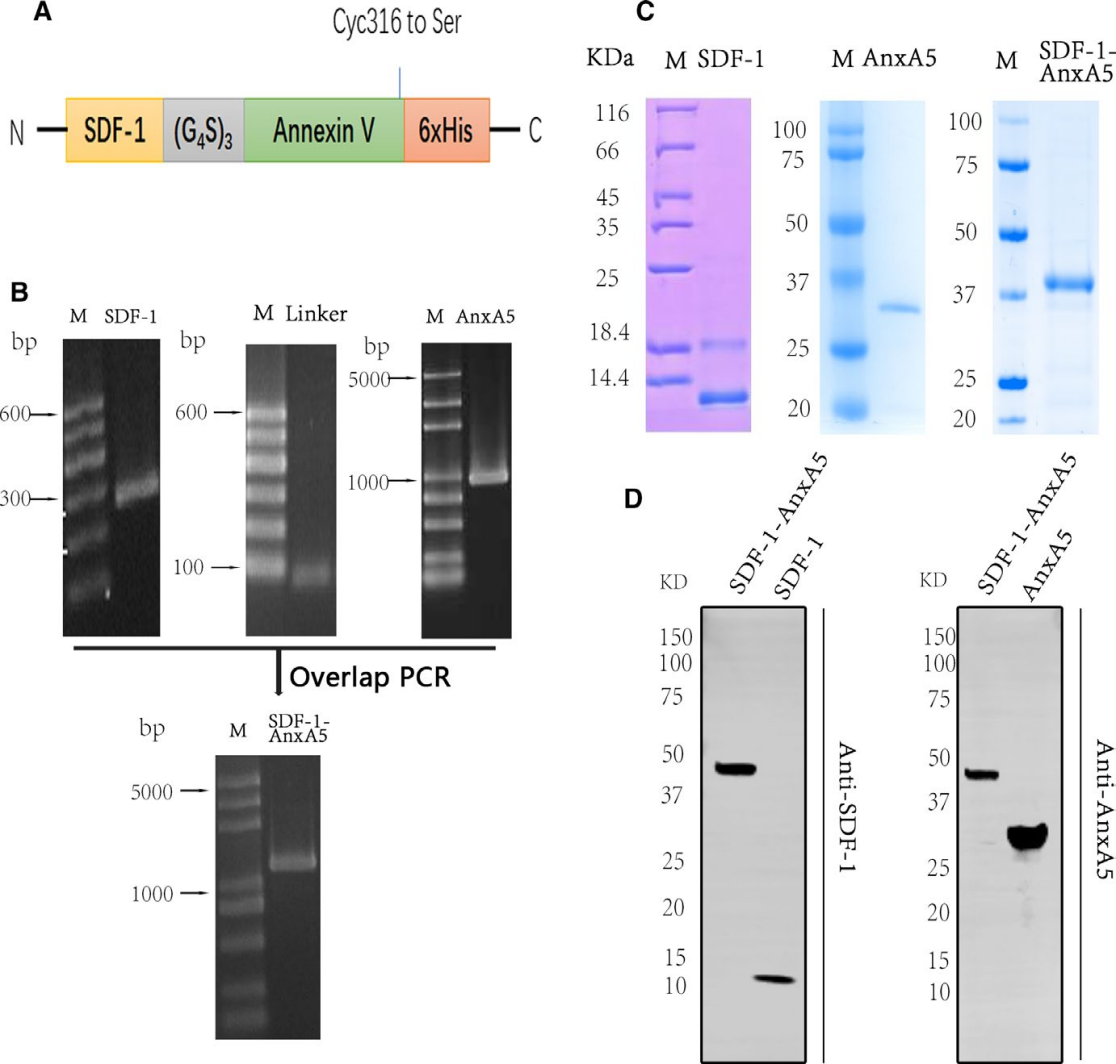
The preparation of native stromal cell‐derived factor‐1 (SDF‐1), AnxA5 and SDF‐1‐Annexin. A, Schematic illustration of SDF‐1‐AnxA5 sequence. Cys316 to Ser is mutation site to avoid mismatching disulfide bones of SDF‐1. B, Agarose gel electrophoresis analysis of SDF‐1, Linker, AnxA5 and SDF‐1‐AnxA5. C, SDS‐PAGE analysis of expression and purification of SDF‐1, AnxA5 and SDF‐1‐AnxA5. D, Native SDF‐1, AnxA5 and SDF‐1‐AnxA5 were identified by Western blot with anti‐SDF and anti‐AnxA5 antibodies

To preserve the bioactivity of SDF‐1, the AnxA5 was linked to the C‐terminal of native SDF‐1 using a linker[(G_4_S)_3_] to separate the two domains (Figure 1A). The endogenous cysteine of AnxA5 at position 316 was mutated to serine to avoid mismatching disulfide bones of SDF‐1 domain. The PCR fragments of SDF‐1, Linker and AnxA5 were shown in Figure 1B. The native SDF‐1, AnxA5 and fusion protein cDNA sequence was inserted into pET‐28a (+) to construct pET‐28a‐SDF‐1‐6xHis, pET‐28a‐AnxA5‐6xHis and pET28a‐SDF‐1‐AnxA5‐6xHis. The recombinant plasmids were identified by restriction endonuclease cleavage and DNA sequencing (Figure S1).

The recombinant proteins were expressed in BL21 (DE3) with the induction of 0.5 mmol/L IPTG. AnxA5 was expressed in the soluble form, while native SDF‐1 and SDF‐1‐AnxA5 were in inclusion bodies. AnxA5 was purified by an affinity chromatography column. The native SDF‐1 and SDF‐1‐AnxA5 inclusion bodies were refolded and purified by on‐column chaperone‐like chemical refolding methods (Figure S2). The Coomassie brilliant blue staining showed that the recombinant proteins were isolated with high purity (Figure 1C). Furthermore, Western blot results showed that the molecular mass of the recombinant proteins was as expected (Figure 1D). Thus, the fusion proteins consisting of SDF‐1 and Annexin V domains were successfully prepared.

### 3.2 SDF‐1‐AnxA5 binds to and activates CXCR4‐ or CXCR7‐mediated signalling

**Figure 2:**
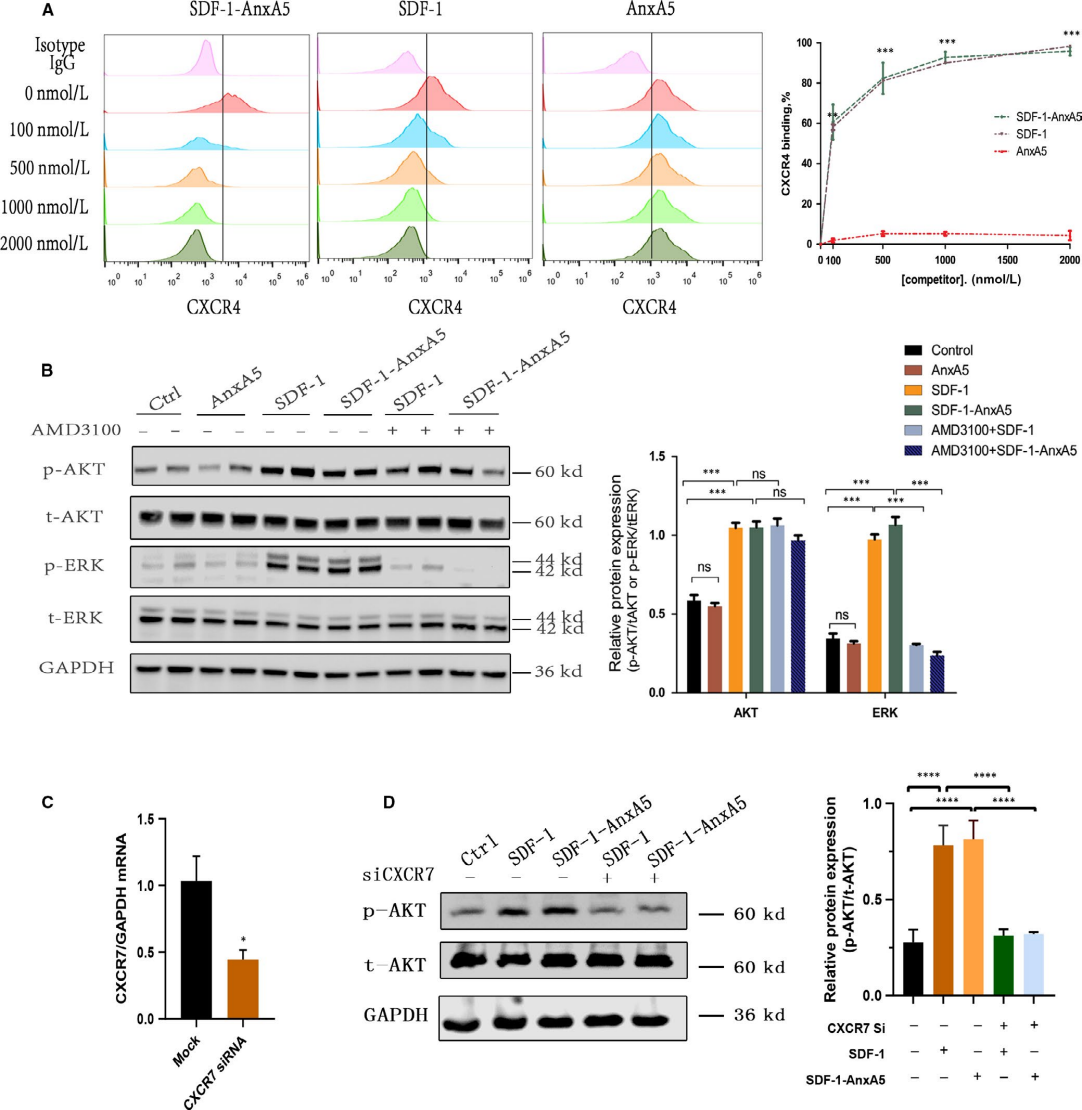
SDF‐1‐AnxA5 binds to and activates CXCR4 and CXCR7 (A) The binding capacity of SDF‐1‐AnxA5 to CXCR4 was examined by pretreatment of MOLT‐4 cells with native SDF‐1‐AnxA5, SDF‐1 and AnxA5 at indicated concentrations. CXCR4 on the cell surface was detected by flow cytometry with anti‐human CXCR4‐PE antibody. The representative flow cytometry and line chart shows that SDF‐1‐AnxA5 and SDF‐1, but not AnxA5, had a dose‐dependent suppression of CXCR4 antibody binding to CXCR4 receptor (n = 3, **P < .01; ***P < .001 vs AnxA5 group). B, The effect of AnxA5, SDF‐1 and SDF‐1‐AnxA5 on p‐ERK and p‐AKT expression with or without the presence of CXCR4 specific antagonist, AMD3100 (n = 3, ***P < .001). C, qPCR analysis of CXCR7 knockdown efficiency by CXCR7 siRNA (n = 3, *P < .05 vs mock). D, The effect of SDF‐1‐AnxA5 and SDF‐1 on p‐AKT expression with or without silencing of CXCR7 (n = 3, ***P < .001; ****P < .0001)

As is well known, the biological effect of SDF‐1 is mediated by binding to the chemokine receptor CXCR4. First, to determine whether SDF‐1‐AnxA5 retains its binding ability to CXCR4, we performed a CXCR4 competition‐based assay using MOLT‐4 cell line that constitutively overexpresses CXCR4. MOLT‐4 cells were incubated with different concentrations of SDF‐1‐AnxA5 fusion proteins to compete with PE‐conjugated anti‐CXCR4 antibodies, and the amount of CXCR4 on the cell surface was measured by flow cytometry. Results showed that SDF‐1‐AnxA5 and native SDF‐1 have a similar binding affinity to CXCR4. No binding to CXCR4 was detected for the AnxA5 (Figure 2A).

Next, we determined whether SDF‐1‐AnxA5 proteins can activate CXCR4‐mediated intracellular signalling. ERK and AKT are known as critical signal transduction associated with SDF‐1 activating CXCR4.^15^ Therefore, we measured the phosphorylation levels of AKT and ERK in MOLT‐4 cells after treatment with different recombinant proteins. SDF‐1‐AnxA5 and native SDF‐1 significantly promoted the phosphorylation of AKT and ERK. In contrast, AnxA5 did not influence p‐AKT or p‐ERK levels compared with control (Figure 2B). Furthermore, when MOLT‐4 cells were pretreated with a CXCR4 selective antagonist AMD3100, both native SDF‐1 and SDF‐1‐AnxA5 failed to induce phosphorylation of ERK, while SDF‐1 or SDF‐1‐AnxA5‐activated p‐AKT did not change much (Figure 2B). In addition to CXCR4, CXCR7 is another functional receptor for SDF‐1. Chen et al found that SDF‐1 could activate p‐ERK but not p‐AKT via CXCR4 mediation in cardiac stem cells, while SDF‐1‐induced p‐AKT could be mediated by CXCR7.^16^ Therefore, we investigated the role of silencing of CXCR7 in SDF‐1 signalling. Silencing of CXCR7 abolished the effect of SDF‐1 or SDF‐1‐AnxA5 on the phosphorylation of AKT (Figure 2C‐D). These experiments indicate that SDF‐1 domain of SDF‐1‐AnxA5 can trigger CXCR4‐ and CXCR7‐associated signalling pathway.

### 3.3 SDF‐1‐AnxA5 promotes chemotaxis and endothelial cell in vitro angiogenesis

**Figure 3:**
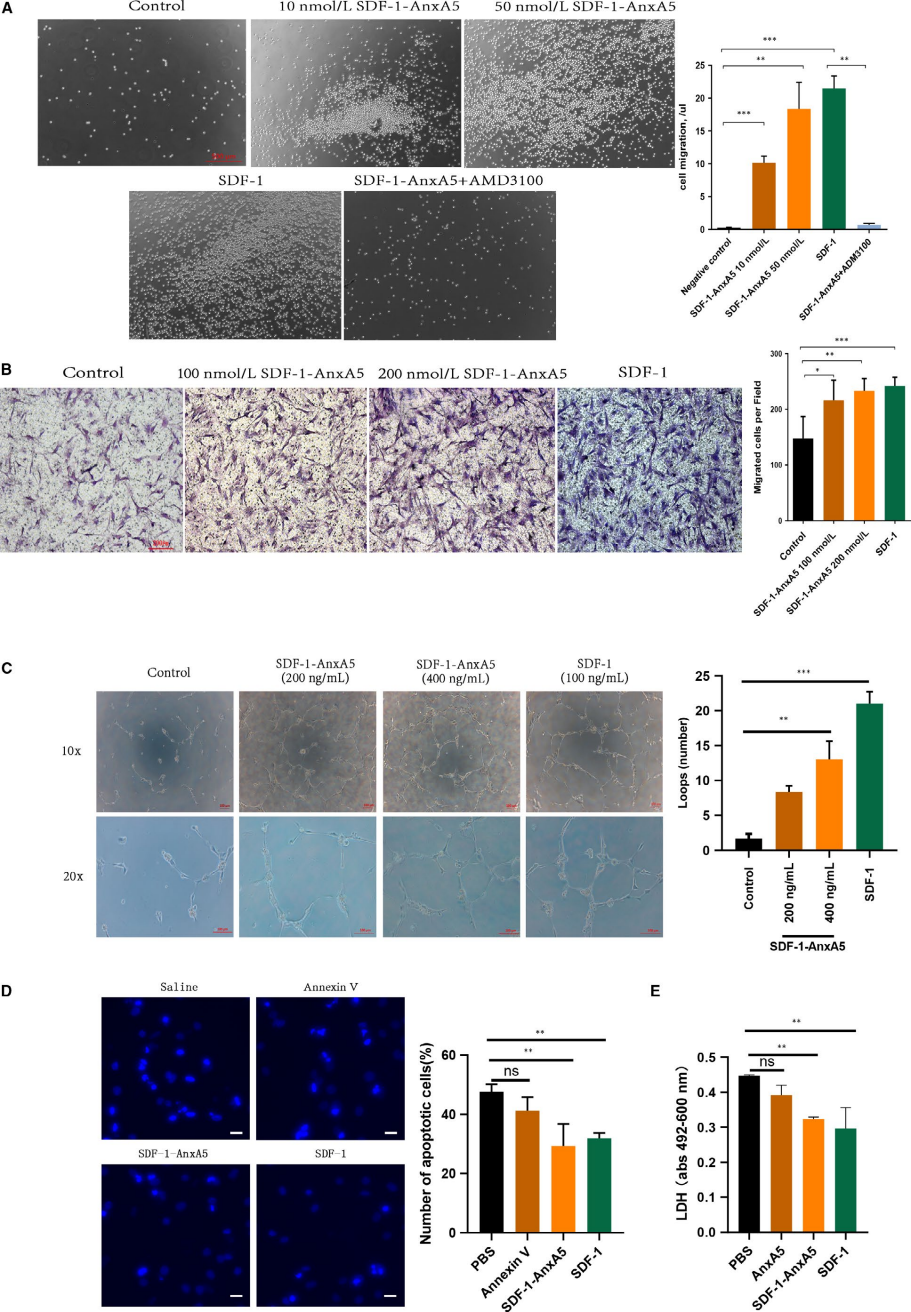
SDF‐1‐AnxA5 promotes chemotactic response, angiogenesis and cell survival. A, SDF‐1‐AnxA5 triggered the migration of MOLT‐4 cells through Transwell membrane. AMD3100 blocked the chemotactic effect of SDF‐1‐AnxA5. The numbers of migrated cells were calculated by flow cytometry (n = 3, **P < .01; ***P < .001 vs control). B, Bone marrow mesenchymal stem cell migration were stimulated by SDF‐1‐AnxA5 or SDF‐1 (n ≥ 3, *P < .05; **P < .01; ***P < .001 vs control). C, The in vitro tube formation of human umbilical vein endothelial cells. The representative image displays that tube formation of human umbilical vein endothelial cell increased with SDF‐1‐AnxA5 treatment in Matrigel matrix (n = 3, **P < .01; ***P < .001 vs Control). D, The apoptosis of neonatal rat ventricular cardiomyocytes was determined by Hoechst 33342 staining (n = 3, **P < .01 vs PBS scale bar = 50 µm). E, The supernant lactic dehydrogenase (LDH) levels were reduced in SDF‐1‐AnxA5 and SDF‐1 group (n=3, **P<.01 vs PBS).

To study the biological function of SDF‐1‐AnxA5, we used a Transwell system to examine the chemotaxis of SDF‐1‐AnxA5. As shown in Figure 3A, SDF‐1‐AnxA5 induced a dose‐dependent chemotactic response of MOLT‐4 cells (control vs SDF‐1‐AnxA5 10 nmol/L group: 0.26 ± 0.04/μL vs 10.1 ± 0.57/μL, *P* < .001, Figure 3A). Furthermore, AMD3100 blocked the SDF‐1‐AnxA5‐mediated migration of MOLT‐4 cells, which implied that the SDF‐1 domain interaction with CXCR4 is responsible for the chemotaxis of SDF‐1‐AnxA5. Previous studies showed that the chemotactic effect of SDF‐1 on the mesenchymal stem cells (MSCs) contributes to cardiac repair after myocardial infarction.^2^ Thus, we tested whether SDF‐1‐AnxA5 can promote the migration of MSCs. The Transwell assay revealed that 100 nmol/L SDF‐1‐AnxA5 and native SDF‐1 significantly enhance migration of MSCs through the Transwell chamber than control (Figure 3B).

Neovascularization is an essential mechanism underlying myocardial repair. SDF‐1 is also known as a major angiogenic protein.^17^ To determine whether SDF‐1‐AnxA5 have the proangiogenic capability, we performed an endothelial cell in vitro tube formation assay which is a powerful in vitro method to screen angiogenic factors.^18^ We found that compared with control, SDF‐1‐AnxA5 and native SDF‐1 augmented the HUVEC tube formation on Matrigel matrix (Figure 3C). The results indicate that SDF‐1‐AnxA5 induces the proangiogenic effect.

Stromal cell‐derived factor‐1 serves as a cardioprotective protein by promoting cardiomyocyte survival.^5^, ^19^ Therefore, we further evaluated the anti‐apoptotic role of SDF‐1‐AnxA5 in hypoxia‐treated cardiomyocytes. Neonatal rat ventricular cardiomyocytes were cultured in oxygen and glucose‐deprived environment. The Hoechst 33342 staining displayed that SDF‐1 and SDF‐1‐AnxA5 but not Annexin V significantly decreased the number of apoptotic cells by 39% and 33%, respectively (Figure 3D). Likewise, the supernatant lactic dehydrogenase was lower in SDF‐1‐AnxA5 than vehicle (*P* < .01, Figure 3E). These data revealed that SDF‐1‐AnxA5 have an anti‐apoptotic action on cardiomyocytes.

### 3.4 SDF‐1‐AnxA5 detects and binds to dead cells in vitro

**Figure 4:**
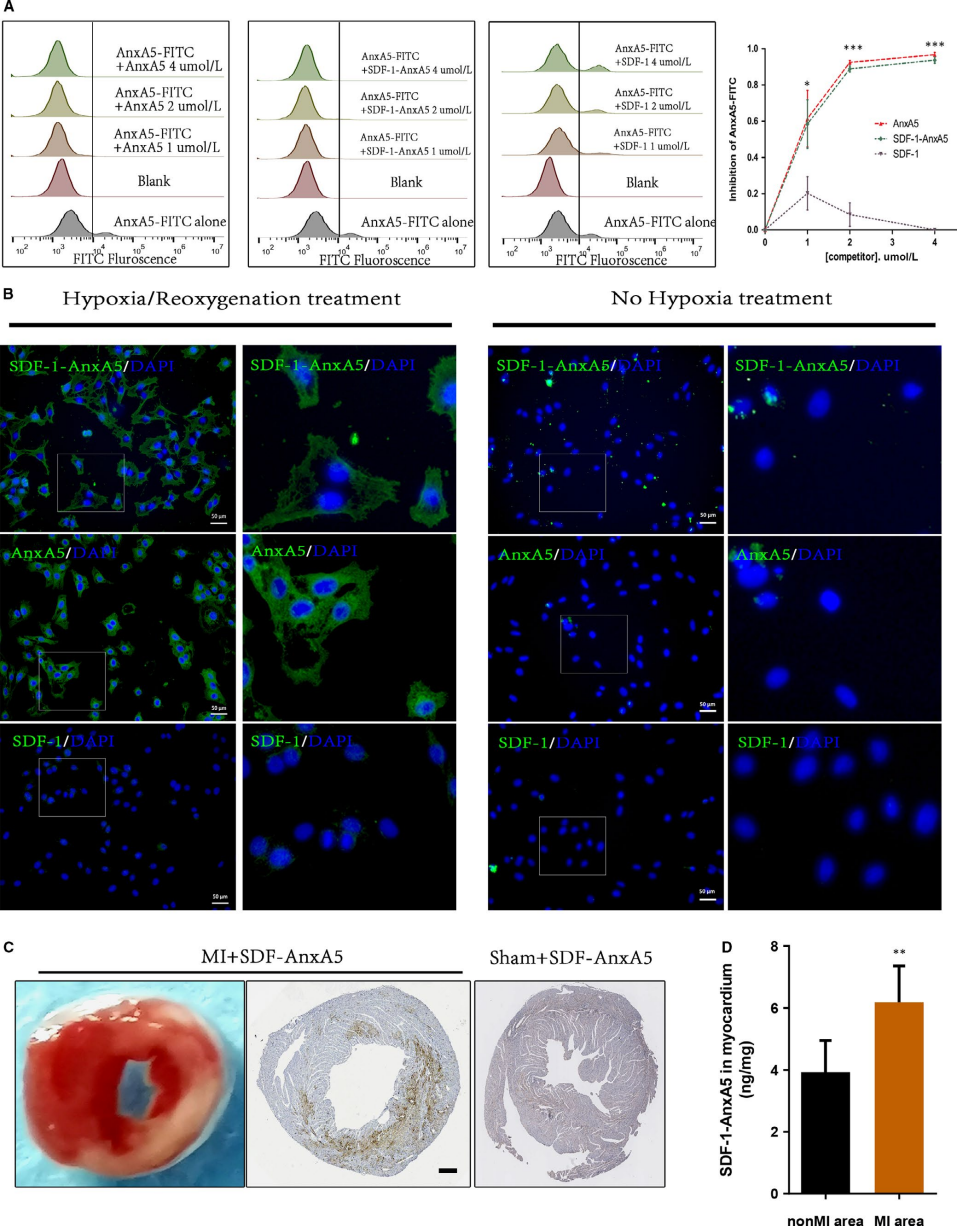
SDF‐1‐AnxA5 identifies and binds to dead cells. A, Membrane binding of SDF‐1‐AnxA5. Proteins were added at the indicated concentrations along with commercial AnxA5‐FITC and apoptosis‐induced H9C2 cells. The representative flow cytometry and line chart shows that SDF‐1‐AnxA5 and AnxA5, but not SDF‐1, could compete with commercial AnxA5‐FITC for binding death cells (n ≥ 3, *P < .05; ***P < .001). B, The immunofluorescent staining for 6xHis‐tag antibody. SDF‐1‐AnxA5(400 ng/mL), AnxA5 (300 ng/mL) or SDF‐1(100 ng/mL) were incubated with H9C2 cells with (right) or without (left) the treatment of hypoxia. C, The representative transverse cardiac section shows infarcted area (TTC negative area) stained by 1%TTC and immunohistochemical staining against anti‐6xHis tagged proteins displays the accumulation of SDF‐1‐AnxA5 administered via tail vein in the infarcted area. Scale bar represents 400 µm. D, the SDF‐1‐AnxA5 concentrations in ischaemic and non‐ischaemic area were measured by ELISA (n = 3, **P < .01 [paired *t* test])

Next, we determined whether SDF‐1‐AnxA5 could in vitro bind to cells with exposed PS, one of the hallmarks of dead cells.^10^ H9C2 cardiomyocytes were treated with hypoxia to induce cell death. The affinity of proteins to be tested for dead cells was determined by competition assay against commercial FITC‐Annexin V. The flow cytometry showed that after hypoxia treatment, about 11.7% of H9C2 cells exposed PS detected by Annexin V‐FITC (Figure S3). Then, SDF‐1‐AnxA5 and AnxA5 displayed a similar dose‐dependent suppression of Annexin V‐FITC binding to dead cells, whereas native SDF‐1 failed to influence Annexin V‐FITC binding to the dead cells (Figure 4A). Furthermore, we examined the specificity of SDF‐1‐AnxA5 binding to dead cells. Immunofluorescence staining for 6xHis‐tagged protein revealed that SDF‐1‐AnxA5 and AnxA5 could specifically bind to hypoxia‐injured cells but not normal cells (Figure 4B). Taken together, the data indicate the highly sensitive and specific binding ability of SDF‐1‐AnxA5 to dead cells.

### 3.5 Intravenous SDF‐1‐AnxA5 accumulates at ischaemic myocardium

The above in vitro experiments reveal that SDF‐1‐AnxA5 can identify and bind to dead cells. We next testified whether SDF‐1‐AnxA5 via intravenous injection could accumulate at the cardiac ischaemic area. We established a mouse MI model by the ligation of LAD. The mice were injected SDF‐AnxA5 proteins via tail vein at 2 days after MI. The infarcted area was shown by 1% 2,3,5‐triphenyltetrazolium chloride staining. The specific accumulation of SDF‐1‐AnxA5 in the ischaemic myocardium was detected by immunohistochemistry. An anti‐polyhistidine antibody was used to distinguish exogenous SDF‐1‐AnxA5 from endogenous SDF‐1. As Figure 4C shows, the enhanced accumulation of SDF‐1‐AnxA5 was observed in the infarcted myocardium but not in sham‐operated animals. In addition, the enzyme‐linked immunosorbent assay revealed that SDF‐1‐AnxA5 concentrations were significantly higher in the ischaemic area than the non‐ischaemic area (Figure 4D). Thus, SDF‐1‐AnxA5 proteins via intravenous administration can efficiently accumulate at ischaemic myocardium.

### 3.6 SDF‐1‐AnxA5 preserves cardiac function after myocardial infarction

**Figure 5:**
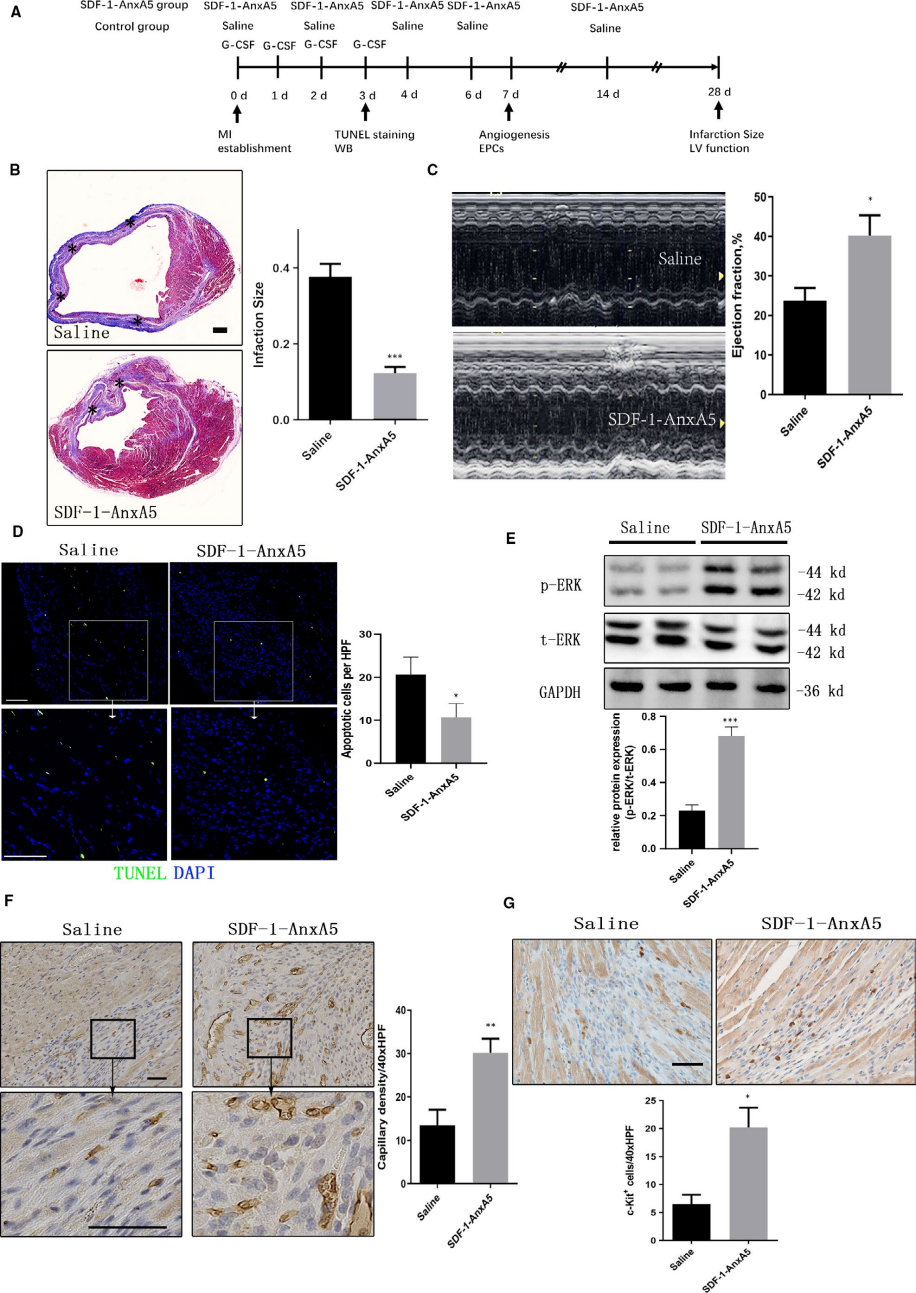
SDF‐1‐AnxA5 treatment reduces infarcted size and preserves cardiac function after myocardial infarction. A, Animal study protocol. B, Representative images (left) of Masson's trichrome staining for cardiac sections. SDF‐1‐AnxA treatment significantly reduced the proportion of scar tissue in the left ventricle 28 d after myocardial infarction. The area where black asterisks are indicates infarcted ones (right; n = 5, ***P < .001 vs control; scale bar = 200 µm). C, Representative M‐mode echocardiography (left). Ejection fraction 28 days after myocardial infarction was improved in SDF‐1‐AnxA5 group compared with control (right; n = 5, *P < .05 vs control). D, Apoptotic cells were detected via TUNEL staining at 3 d after MI (upper). The numbers of TUNEL‐positive nuclei per high field of view were calculated and analysed (below; n = 3, *P* < .05 vs control; scale bar = 100 µm). E, Western blots with heart lysates 3 days after MI and treated with saline or SDF‐1‐AnxA5 (n = 3, ***P < .001). F, Immunohistochemical analysis (left) against PECAM‐1, an endothelial marker, in cardiac sections from mice 7 d after myocardial infarction. The SDF‐1‐AnxA5 treatment significantly increased the density of capillary vessels in the ischaemic area (right; **P < .01 vs control; top bar = 50 µm). G, Representative images of immunohistochemical staining for c‐kit^+^ of cardiac sections from mice 7 d after myocardial infarction (upper). Quantification of c‐kit^+^ cells in the ischaemic area is shown (below; n = 5, *<0.05 vs control; scale bar = 50 µm)

We next explored the effect of SDF‐1‐AnxA5 on the post‐infarction myocardial repair. After establishing the MI model, we administered SDF‐1‐AnxA5 via tail vein injection immediately and 2, 4, 6 and 14 days. Meanwhile, G‐CSF was administered intraperitoneally for four consecutive days to mobilize endogenous stem/progenitor cells from bone marrow^20^ (Figure 5A).

The infarcted area was assessed by Masson’ trichrome staining. In the SDF‐1‐AnxA5‐treated group, the infarction size at 28 days after MI was significantly reduced compared to control group (12.4%±1.6% vs 37.8%±3.3%, *P* < .001, [infarction size as the ratio of infarct area/total ventricular area], Figure 5B). The cardiac function was evaluated by echocardiography at 28 days after MI. We found that SDF‐1‐AnxA5 improved left ventricular ejection fractions after MI by 69% (SDF‐1‐AnxA5 group vs control, 40.2% ± 5.2% vs 23.8% ± 3.2%, *P* < .05, Figure 5C).

To investigate the underlying mechanism by which SDF‐1‐AnxA5 confers cardioprotection, we firstly examined the number of dead cells after MI. Apoptotic cells marked by terminal deoxynucleotidyl transferase‐mediated dUTP‐biotin nick end labelling (TUNEL) assay were observed in both control and SDF‐1‐AnxA5 groups after MI. SDF‐1‐AnxA5 treatment reduced the number of TUNEL‐positive nuclei compared with saline (20.7 ± 4.0 vs 10.7 ± 3.2/HFP, *P* = .03, Figure 5D). In the harvested heart cell lysates, we observed increased levels of p‐ERK in SDF‐1‐AnxA5 after MI (Figure 5E). Then, we investigated the influence of SDF‐1‐AnxA5 administration on vascularization of ischaemic area. Immunohistochemical staining for PECAM‐1, an endothelial cell marker, revealed that in SDF‐1‐AnxA5‐treated group, the capillary density was higher at 7 days compared with the control group (30.2 ± 3.3 vs 13.5 ± 3.5/HFP, *P* < .01, Figure 5F). As it is suggested that the SDF‐1‐mediated recruitment of endothelial progenitor cells (EPCs) plays a critical role in the angiogenesis, we examined whether intravenous administration of SDF‐1‐AnxA5 can enhance the recruitment of endogenous EPCs. Considering that c‐kit is a marker of EPCs,^21^, ^22^, ^23^ we used a c‐kit antibody to detect EPCs in the infarcted area at 7 days after MI. The results showed that the counts of c‐kit^+^ cells were higher in SDF‐1‐AnxA5‐treated group than control group (20.2 ± 3.5 vs 6.5 ± 1.8/40xHPF, *P* < .05), indicating that SDF‐1‐AnxA5 could enhance the enrichment of EPCs in the ischaemic area (Figure 5G). Taken together, these data revealed that the intravenous administration of SDF‐1‐AnxA5 can enhance cell survival, EPC recruitment and angiogenesis.

## 4 DISCUSSION

In the present study, we successfully constructed a bifunctional fusion protein consisting of SDF‐1 and Annexin V domains. The in vitro assays demonstrated that SDF‐1‐AnxA5 can bind to and activate chemokine receptor CXCR4 to induce chemotactic response and angiogenesis; SDF‐1‐AnxA5 also possesses the ability to identify and bind to dead cells. The intravenous administration of SDF‐1‐AnxA5 contributes to enhanced angiogenesis, decreased the infarcted size and preserved cardiac function after MI.

SDF‐1 confers cardioprotection in multiple aspects after MI. Firstly, SDF‐1 protects myocardium from ischaemia‐induced apoptosis to limit the expansion of infarcted size.^19^ Furthermore, SDF‐1 can also promote angiogenesis in infarcted area to facilitate revascularization. Meanwhile, our and other previous studies have found that beyond its role in mobilization and homing of bone marrow cells, SDF‐1/CXCR4 axis can promote bone marrow‐derived cells to give rise to cardiomyocyte‐like phenotype.^24^, ^25^ Thus, the importance of SDF‐1/CXCR4 axis in myocardial repair has attracted considerable attentions.

Several studies have attempted to administer exogenous SDF‐1 to maintain the high concentrations of local SDF‐1 in ischaemic myocardium. These approaches included intramyocardial injection of SDF‐1 proteins or its derivatives,^26^, ^27^, ^28^ SDF‐1 viral^29^ or non‐viral gene^30^ and SDF‐1‐overexpressing MSCs^8^ or cardiac fibroblasts.^31^ Although these strategies have been shown to increase SDF‐1 concentrations in the infarcted area and preserve cardiac function, the difficulties in operation and controllability hinder their clinical application. Thus, it is necessary to develop a simple and controllable approach to delivery SDF‐1 to the ischaemic area.

AnxA5 is a calcium‐dependent phospholipid binding protein that has a nanomolar affinity for binding to externalized PS. Myocardial infarction results in massive cardiomyocyte death that is characterized by PS externalization on the cell membrane. Cardiomyocyte PS exposure after an ischaemic insult of only 5 minutes can be detected by AnxA5.^32^ Recently, AnxA5‐related imaging reagents have been developed to identify ischaemic area.^33^ Meanwhile, Wakabayashi et al^34^ have found that injured myocardium can still uptake ^99m^Tc‐AnxA5 2 weeks after myocardial ischaemia. These evidences suggest that AnxA5 can be a potential carrier to deliver functional proteins to ischaemic tissue at least during two weeks post‐MI. Van Rite et al^35^ have reported that a fusion protein consisting of AnxA5 and an antitumour protein domains is able to recognize and bind to exposed PS by breast cancer cells and enhance the antitumour effect in vitro. Consistent with previous findings, our experiments demonstrated that SDF‐1‐AnxA5 could specifically recognize and bind to H9C2 cells treated with hypoxia. Furthermore, the present study firstly demonstrates in vivo that AnxA5‐based delivery system helps to enhance the local concentration of functional proteins (such as SDF‐1) in ischaemic area.

The interaction of SDF‐1 with CXCR4 or CXCR7 on the cell surface is critical to produce a biological effect. The structure study has shown that SDF‐1 activation is dependent on the first eight N‐terminal amino acid residues that participate in binding and activation of the receptor.^36^ In addition, Ziegler et al^3^ have reported that GPVI‐SDF‐1 fusion protein (SDF‐1 domain at the C‐terminal of the fusion protein) has dramatically weakened binding affinity for CXCR4 receptor and functional activity. Therefore, we designed the SDF‐1 domain at the N‐terminal of the fusion protein. Our data showed that SDF‐1 domain retains the capability of interacting and activating CXCR4 and CXCR7 receptor (Figure 2). The in vitro experiments also demonstrated that SDF‐1‐AnxA5 could induce a chemotactic response of MOLT‐4 and MSCs, even though it required a higher concentration of SDF‐1‐AnxA5 to promote migration of MSCs, which may be due to the down‐regulation of surface CXCR4 expression on MSCs during in vitro culture expansion.^37^

Apoptosis of massive cardiomyocytes results in adverse myocardial remodelling following MI. SDF‐1‐mediated cell survival has been well characterized.^5^, ^19^ In agreement with previous studies, our in vitro and in vivo experiments showed that the cell apoptosis after MI could be attenuated by SDF‐1‐AnxA5 or SDF‐1, which may contribute to the myocardial repair.

Another important indicator of myocardial repair is the neovascularization in infarcted myocardium. It is well known that SDF‐1 itself is also a potent angiogenic cytokine. In the present study, we performed an in vitro HUVEC tube formation assay to assess the proangiogenic potential of SDF‐1‐AnxA5. These results are consistent with previous findings that SDF‐1 can induce tubulogenesis of HUVECs.^17^ Meanwhile, SDF‐1 treatment attracts c‐kit^+^ endothelial progenitor cells that participate in angiogenesis.^38^ We also observed the enhanced recruitment of c‐kit^+^ cells were accompanied by increased capillary density in the SDF‐1‐AnxA5 group. Hence, SDF‐1‐AnxA5 may increase vascularization to mitigate myocardial injury.

In summary, our study provides a novel delivery strategy that AnxA5 can be used as an anchor to carry functional protein to the injured myocardium. The systemic administration of bifunctional SDF‐1‐AnxA5 effectively attenuates cell apoptosis, enhances vascularization, reduces infarcted size and preserves cardiac function after MI. Therefore, it may be a promising tool to facilitate the chemokine‐based therapy for myocardial infarction.

## CONFLICT OF INTEREST

The authors confirm that there are no conflicts of interest.

## AUTHOR CONTRIBUTIONS

HFY, CM and LXJ designed the study. HFY, XTL, LWH, ZZG and LJL performed experiments. HFY, CL, LCM and LYB analysed the data. HFY, LCM and XD interpreted the results. PY and WYB carried out critical revision of the manuscript for important intellectual content.
