# Supplementary material for: The bifunctional SDF‐1‐AnxA5 fusion protein protects cardiac function after myocardial infarction
Source: J Cell Mol Med. 2019 Aug 30;23(11):7673–84. doi: 10.1111/jcmm.14640 (PMC6815779; doi:10.1111/jcmm.14640)
Supplement: Supplementary file 1 [file JCMM-23-7673-s001.docx]

**Supplemental information**

**Reagents and antibodies.** PrimeSTAR HS DNA Polymerase, QuickCut^TM^ Nco I, QuickCut^TM^ BamH I and DNA Ligation Kit Ver2.1 were purchased from Takara China. E. coli cell strain BL21 (DE3), TIANprep Rapid Mini Plasmid Kit were purchased from TIANGEN Biotech. M9CA minimum medium, vitamin B1, Isopropyl β‑D‑1‑thiogalactopyranoside (IPTG), PMSF, imidazole and guanidine hydrochloride (Gn-HCL) were purchased from Sangon Biotech. Kanamycin, Dithiothreitol (DTT), Triton X-100, β-cyclodextrin, glutathione reductase, and oxidized glutathione were purchased from Sigma-Aldrich. Ni-NTA agarose was purchased from Qiagen. Econo-Pac gravity-flow column was purchased from Bio-Rad. ToxinSensor^TM^ endotoxin removal kit, ToxinSensorTM Chromogenic LAL Endotoxin Assay Kit, ExpressPlus PAGE Gel, recombinant Granulocyte colony-stimulating factor (G-CSF) and 6xHis Tag ELISA Detection Kit were purchased from Genscript. Transwell chambers and Matrigel Basement Membrane Matrix (reduced growth factor) were purchased from Corning. BCA protein Assay kit, Crystal violet and RIPA buffer were purchased from Beyotime. SDF-1 antibody, AnxA5 Antibody, 6xHis-tag antibody phosphor-AKT antibody, Akt(pan)(11E7) antibody, phospho-Erk1/2 antibody, Erk1/2 antibody and c-kit antibody were purchased from Cell Signaling Technology. PECAM-1 antibody was purchased from R&D system. PE mouse anti-Human CD184 and PE mouse IgG2α, κ Isotype control were purchased from BD Biosciences. Annexin V-FITC apoptotic cell detection kit was purchased from KeyGEN Biotech. DeadEnd™ Fluorometric TUNEL System was purchased from Promega.

**Inclusion body protein renaturation**. The inclusion bodies were firstly dissolved by denaturing buffer containing 6M Gn-HCL. Soluble inclusion bodies were applied to Ni-NTA agarose that had been pre-equilibrated with denaturing buffer. Then, the mixture was loaded into a Econo-Pac gravity-flow column. The chromatography steps were done under gravity. The protein on-column refolding was performed through adding detergent buffer containing 1%Triton X-100(v/v) and 5mM DTT and then oxidized buffer containing 5mM β-cyclodextrin, 1mM reduced glutathione and 0.5mM oxidized glutathione to the column. In the next step, the column was washed with wash buffer containing 500mM NaCl. The refolded proteins were eluted with elution buffer containing 1M imidazole. For the purification of AnxA5, the supernatant containing AnxA5 was added to Ni-NTA agarose Ni-NTA agarose. Then, the column was washed with wash buffer and eluted with elution buffer. All these elutions were desalted by dialysis against phosphate solution overnight. Furthermore, the endotoxin of protein solutions was removed using ToxinSensor^TM^ endotoxin removal kit according to the manufacturer’s protocol. After removal of endotoxin, the endotoxin levels of the protein solution were less than 0.1EU/ug.

**Western blot**. Purified protein solutions were mixed with SDS-PAGE protein loading buffer, heated to 95℃ for 5minutes, separated on an ExpressPlus PAGE Gel and the proteins were then transferred onto a PVDF membrane. The membrane was blocked using 5% skim milk powder in TBST and then incubated with the indicated concentrations of primary antibody at 4℃ overnight. After washing with TBST, the membrane was incubated with HRP-labeled secondary antibodies for 2 hours at RT. The membrane was detected by a Bio-Rad ChemiDox MP system.

Cell culture. MOLT-4 and H9C2 (obtained from ATCC) cells were cultured in DEME supplemented with 10% fetal bovine serum (FBS). Mouse bone marrow mesenchymal stem cells (obtained from Cyagen biosciences) were cultured in OriCell C57BL/6 mouse bone marrow mesenchymal stem cell complete medium (Cyagen). human umbilical vein endothelial cells (HUVECs) were cultured in complete endothelial cell growth medium (AllCells). The cells were maintained at 37℃ in a humidified 5% CO2 incubator.

**Determination of p-Akt, Akt, p-Erk1/2 and Erk1/2 protein expression.** MOLT-4 cells were starved with serum-free medium RPMI-1640 for 2 hours and harvested in the 1.5ml EP tubes. Ten minutes after treatment, cells were harvested and lysed in RIPA buffer. The proteins were collected for detection of total p-Akt, total Akt, p-Erk1/2 and total Erk1/2 by Western blot.

**Neonatal rat cardiomyocytes (NRVM) isolation.** Single cardiac myocytes from the hearts of 2-3-day-old Sprague Dawley rats were isolated by digestion with collagenase. Briefly, ventricles from neonatal rats were cut into pieces and then dissociated in calcium-free HBSS containing 0.05% trypsin (Gibco) and 0.05% collagenase II (Gibco). Digestion was performed at 37°C in eight to ten 5-min steps, collecting the supernatant to FBS after each step. The collected cells were passed through a cell strainer (100µm, BD Falcon) and then centrifuged and resuspended in DMEM supplemented with 10% FBS and with 100µM 5-bromodeoxyuridine (BrdU, Sigma). the cells were seeded onto 100-mm plastic dishes for 1.5h at 37°C in 5% CO_2_ and humidified atmosphere. The supernatant was then collected and plated onto 12 or 24-well dishes. The next day, the medium was changed by DMEM with 10%FBS and 100µM Brdu.

**Oxygen and glucose deprivation model and Hoechst 33342 staining.** The cell medium was replaced with glucose and serum-free DMEM(Gibco) containing Annexin V(300ng/ml), SDF-1(100ng/ml) or SDF-1-AnxA5(400ng/ml). Then the NRVM were cultured in an oxygen-deficient environment (1%O_2,_ 5%CO2, and 94%N2) for 8 hours and reoxygenation for another 16 hours. The cells of each group were stained with 1µg/ml Hoechst 33342 for 10 min. cell apoptosis was characterized by chromatin condensation and nuclear fragmentation. The apoptosis index was expressed as a ratio of apoptotic to total cells.

**Immunofluorescence**. H9C2 cells were inoculated into 24-well plates and underwent hypoxia (5% O2, 5%CO2, and 90%N2) and reoxygenation treatment. Cells were washed with PBS and incubated with different recombinant proteins in the presence of calcium for 10 minutes at 4℃. After washed, cells were fixed with 4% paraformaldehyde (containing Ca^2+^) for 30 minutes at RT, permeabilized with 0.5%Triton X-100 for 10 minutes, and then incubated with 5%goat serum HBST (0.1%Tween in HEPES-buffered solution) for 30 minutes. Anti His-tag antibody was diluted in HBST (1:800) and incubated with the cells overnight at 4℃. On the following day, the cells were washed with HEPES three times and incubated with the anti-rabbit secondary antibody conjugated to Alexa Fluor 488(1:400) for 2 hours at RT. Finally, the cells were stained with DAPI (1:1000) for 5 minutes after secondary antibodies were removed by washing. Cells were observed under a fluorescence microscope.

**Enzyme-linked immune-sorbent assay.** After 2 days of MI, SDF-1-AnxA5 was administered through the tail vein. After 30minutes, hearts were harvested and immediately frozen with liquid nitrogen. Heart tissues from the ischemic and non-ischemic area were respectively cut off and lysed in the RIPA by tissue homogenate. The total protein concentrations were determined by BCA protein Assay kit. The concentration of SDF-1-AnxA5 was detected according to the manual of the His-Tag ELISA Detection Kit according to the manufacturer’s protocol.

**The accumulation of SDF-1-AnxA in the infarcted area.** After 2 days of MI, SDF-1-AnxA5 was administered through the tail vein. After 30minutes, hearts were harvested and cut in half at the infarcted area. half of the heart was Immunohistochemically analyzed with an anti-His-tagged antibody. The other half of heart was stained with 1% triphenyltetrazolium chloride (TTC). The slices were immersed in 10% formalin. TTC staining negative area indicates infarcted myocardium.

**Histological analysis.** Specimens were fixed in 10% formalin for 48 hours and embedded in paraffin, and 3um-thick sections were cut and mounted on the positively charged glass slides. Apoptosis in the adult heart was assayed with the DeadEnd Fluorometric TUNEL system (Promega). Masson’s trichrome staining was used to assess the scar size. An anti-c-kit antibody was used to stain the c-kit+ cells. An antibody against PECAM-1 was used to evaluated capillary vessel density. The Image-Pro Plus was used to count the number of c-kit+ cells and capillary density.

**Animal echocardiography.** After mice were anesthetized by inhalation of isoflurane, echocardiography was performed using a GE Vivid 7 equipped with a 12-MHz imaging transducer. Measurements were performed at the midpapillary level from well-aligned M-mode images from the parasternal short axis view.


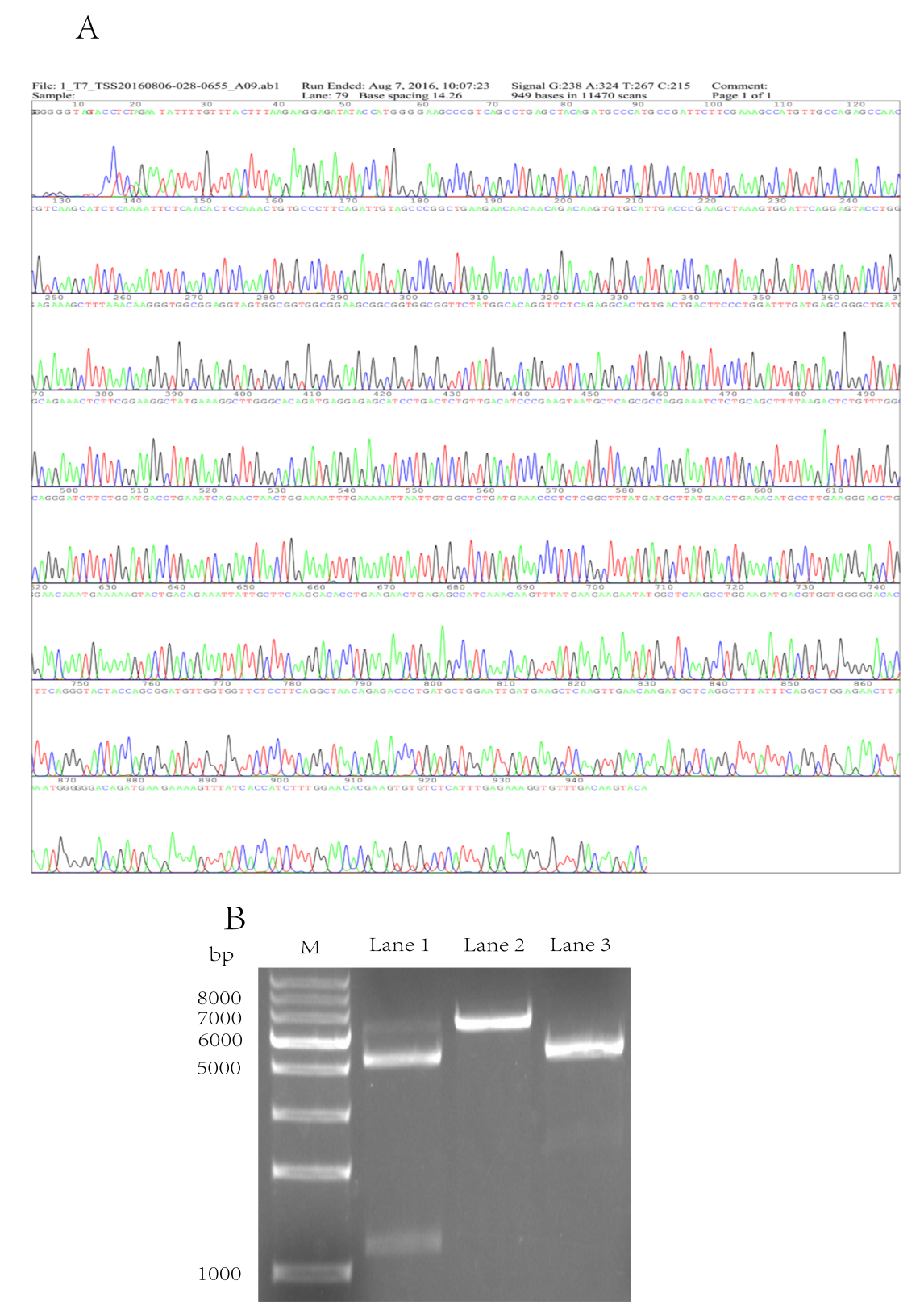


**Supplemental Figure 1.** The verification of SDF-1-AnxA5 fusion gene. (A) SDF-1-AnxA5 fusion gene sequence. (B) Identification of recombinant plasmid pET28a-SDF-1-AnxA5-6xHis digested by *Nco*I and *Bam*HI restriction enzymes. Lane 1, the plasmid doubly digested with *Nco*I and *Bam*HI restriction enzymes; Lane 2, the plasmid digested by only *Bam*HI restriction enzyme; Lane 3, the empty pET28a vector digested by *Bam*HI restriction enzyme.


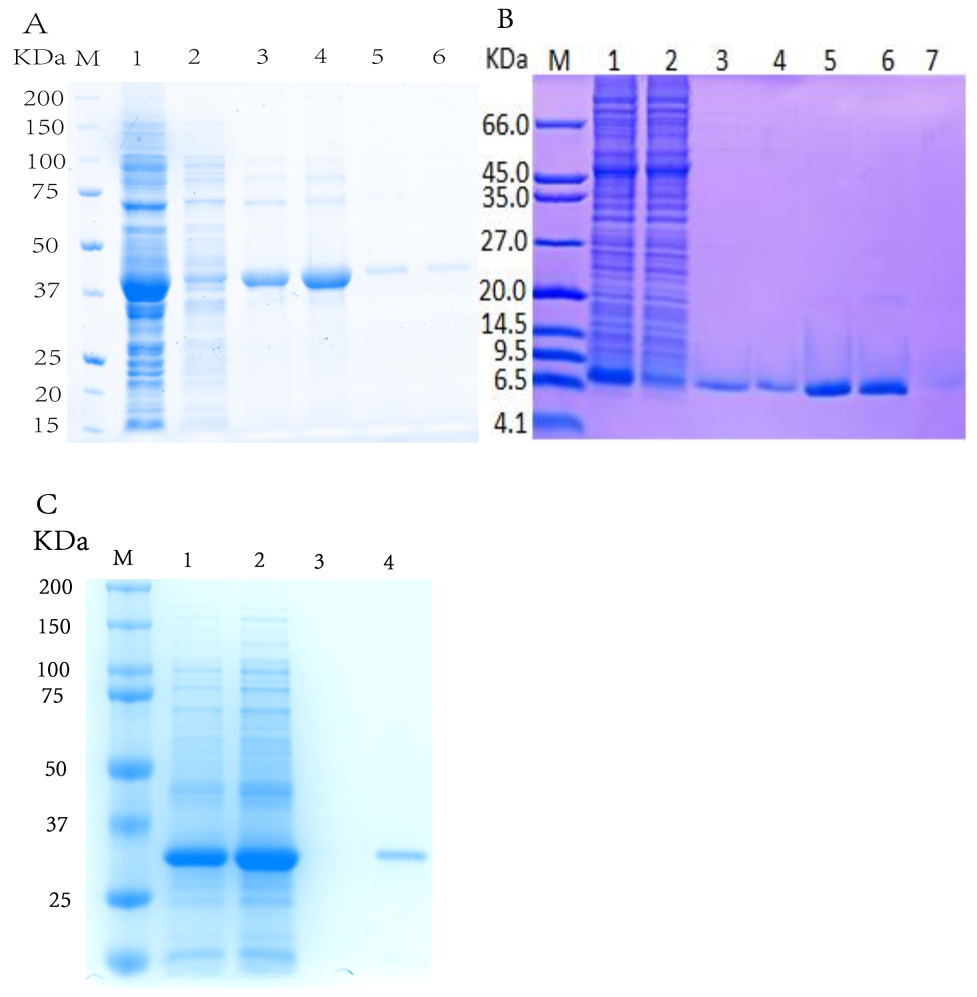
**Supplemental Figure 2.** SDS-PAGE analysis of purification of SDF-1-AnxA5, SDF-1 and AnxA5. (A) the purification of SDF-1-AnxA5. Lane 1, bacterial inclusion body; Lane 2, flowthrough; Lane 3, wash; Lane 4-6, elution 1-3. (B) the purification of SDF-1. Lane 1, bacterial inclusion body; Lane 2, flowthrough; Lane 3, wash; Lane 4-6, elution 1-3. (C) the purification of AnxA5 (10x dilution). Lane 1, flowthrough; Lane 2, the supernatant of bacterial lysates; Lane 3, wash; Lane 3, elution (50mM imidazole); Lane 4, elution (1M imidazole).


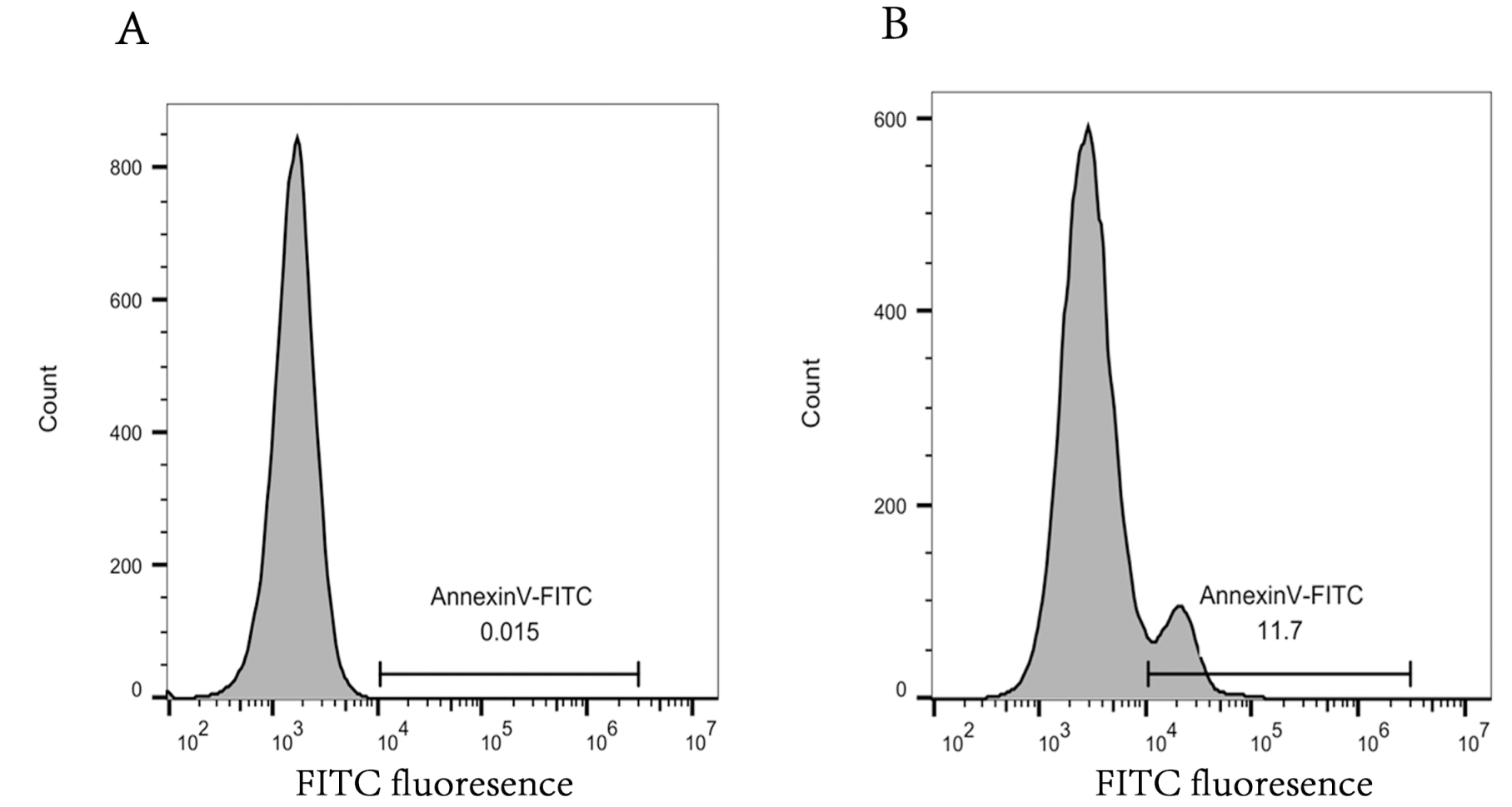


**Supplemental Figure 3.** The exposure of PS in H9C2 cells after the treatment of hypoxia. H9C2 cells underwent normoxia (A) or hypoxia (B) treatment and incubated with commercial AnxA-FITC. The flowcytometry detected the amounts of FITC-positive H9C2 cells.
